# Effects of midgut bacteria in *Hyphantria cunea* (Lepidoptera: Erebidae) on nuclear polyhedrosis virus and *Bacillus thuringiensis* (Bacillales: Bacillaceae)

**DOI:** 10.1093/jisesa/iead009

**Published:** 2023-03-14

**Authors:** Hongjian Chen, Dejun Hao, Changyu Chen, Yuhang Sun, Xiaohang Yu

**Affiliations:** Co-Innovation Center for Sustainable Forestry in Southern China, Nanjing Forestry University, Nanjing 210037, China; College of Forestry, Nanjing Forestry University, Nanjing 210037, China; Co-Innovation Center for Sustainable Forestry in Southern China, Nanjing Forestry University, Nanjing 210037, China; College of Forestry, Nanjing Forestry University, Nanjing 210037, China; Co-Innovation Center for Sustainable Forestry in Southern China, Nanjing Forestry University, Nanjing 210037, China; College of Forestry, Nanjing Forestry University, Nanjing 210037, China; Co-Innovation Center for Sustainable Forestry in Southern China, Nanjing Forestry University, Nanjing 210037, China; College of Forestry, Nanjing Forestry University, Nanjing 210037, China; Co-Innovation Center for Sustainable Forestry in Southern China, Nanjing Forestry University, Nanjing 210037, China; College of Forestry, Nanjing Forestry University, Nanjing 210037, China

**Keywords:** *Hyphantria cunea*, intestinal bacteria, synergistic effect, antagonistic effect, Nuclear Polyhedrosis Virus

## Abstract

*Hyphantria cunea* Drury (Lepidoptera: Erebidae) is a quarantine pest in China that can cause damage to hundreds of plants. As biological control agents, Nuclear Polyhedrosis Virus (NPV) and *Bacillus thuringiensis* Berliner (Bacillales: Bacillaceae) (*Bt*) are commonly used to inhibit the prevalence of *H. cunea*. To investigate the role of midgut bacteria in the infection of NPV and *Bt* in *H. cunea*, we performed a series of tests, including isolating the dominant culturable bacteria in the midgut, eliminating intestinal bacteria, and respectively inoculating the dominant strains with NPV and *Bt* for bioassay. Two dominant bacteria, *Klebsiella oxytoca* Lautrop (Enterobacterales: Enterobacteriaceae) and *Enterococcus mundtii* Collins (Lactobacillales: Enterococcaceae), in the midgut of *H. cunea* were identified, and a strain of *H. cunea* larvae without intestinal bacteria was successfully established. In the bioassays of entomopathogen infection, *K. oxytoca* showed significant synergistic effects with both NPV and *Bt* on the death of *H. cunea.* In contrast, *E. mundtii* played antagonistic effects. This phenomenon may be attributed to the differences in the physico-chemical properties of the two gut bacteria and the alkaline environment required for NPV and *Bt* to infect the host. It is worth noting that the enhanced insecticidal activity of *K. oxytoca* on NPV and *Bt* provides a reference for future biological control of *H. cunea* by intestinal bacteria.

## Introduction

The fall webworm, *Hyphantria cunea* Drury (Lepidoptera: Erebidae), is a polyphagous defoliator native to North America. Since the 1940s, it has been inadvertently introduced to more than 32 countries in Europe and Asia (Cao et al. 2016). Fall webworms can damage ornamental, forest, and fruit trees as well as crops of more than 400 plant species, causing serious ecological and economic losses (Schowalter and Ring 2017, Feng et al. 2021). Owing to its extreme polyphagy, high fecundity, short generation time and easy spread, many countries have listed *H. cunea* as a quarantine pest (Cao et al. 2016, Gong et al. 2020, Wang et al. 2020a). Since it was first found in Dandong City, Liaoning Province, in 1979, this pest has spread to 13 provincial regions in China (Geng et al. 2022).

Management of fall webworms is based on several strategies, including physical removal, biocontrol, insecticides, and traps (Dara 2019, Edosa et al. 2019, Mikami et al. 2020). Application of chemical pesticides has long been considered as the most effective means of pest control; however, the misuse of traditional insecticides can be detrimental to the environment and the health of humans and animals (Huang et al. 2003, Edosa et al. 2019, Mostafiz et al. 2022, Mssillou et al. 2022). Therefore, Nuclear Polyhedrosis Virus (NPV) and *Bacillus thuringiensis* (*Bt*) have been used as alternatives to inhibit the prevalence of the fall webworm (Boucias and Nordin 1977, Saruhan et al. 2014, Khan and Ahmad 2019).

NPV is a baculovirus, a DNA virus that specifically infects arthropods, especially Lepidoptera, Diptera, and Hymenoptera (van Regenmortel et al. 2000). *H. cunea* Nuclear Polyhedrosis Virus (HcNPV) enters the midgut after being ingested by insects; the polyhedrin (a viral protein) then dissolves and releases virions of polyhedron-derived virus (PDV) in the alkaline environment of the midgut (Granados 1978, Wang and Hu 2019). *H. cunea* infected with HcNPV shows symptoms of pale body color, abnormal movement, and fragile body wall, while the epizootics of HcNPV can cause dramatic reductions in highly dense populations of *H. cunea* (Hajek 1997).

*Bt* is a gram-positive bacterium that can produce parasporal crystals during the sporulation stage (Bravo et al. 2011). These crystals, also known as insecticidal crystal proteins (ICP), are enzymatically hydrolyzed into toxins in the intestine of insect larvae under alkaline conditions, causing the death of insects (Ellis et al. 2002). To date, many strains with high toxicity to many species of Lepidoptera, Coleoptera, and Diptera have been isolated (Sayyed et al. 2004). In *Dendrolimus superans* (Lepidoptera: Lasiocampidae), the symptoms of refusal to eat, decreased activity, and soft body wall in the larvae gradually appear after feeding on *Bt* (Wang et al. 1996).

During long-term co-evolution, insects have formed a close symbiotic relationship with a variety of microorganisms, including viruses, bacteria, fungi, protozoa, nematodes, and so on (Engel and Moran 2013, Xu et al. 2019). Microorganisms colonize exoskeletons, intestines, blood cavities, and even cells of insects, but they are most abundant in the intestines (Douglas 2015). Being an important site for food digestion, nutrient absorption, and metabolism, the intestine of an insect is divided into three parts: foregut, midgut, and hindgut (Chapman et al. 2013). The midgut is where food digestion and absorption take place (Chapman et al. 2013, Holtof et al. 2019). In Lepidoptera, the midgut is alkaline, while other segments are nearly neutral (Chapman et al. 2013). The alkaline environment of the midgut facilitates not only the digestion of food but also the colonization by HcNPV and *Bt*. The intestinal microflora of insects is usually composed of bacteria, fungi, protozoa, and archaea, among which bacteria are the most dominant (Schloss et al. 2006). The common intestinal symbiotic bacteria in insects are Proteobacteria, Bacteroidetes, Firmicutes, Actinobacteria, Verrucomicrobia, and Spirochetes (Chen et al. 2020). Intestinal bacteria in insects display a variety of functions in nutrition metabolism, growth, and development, pathogen resistance, and physiological activities (Engel and Moran 2013). Insects can also protect their body against parasites and pathogens through colonization resistance of intestinal symbiotic bacteria (Engel and Moran 2013, Oliver and Perlman 2020, Bai et al. 2021). *Bombyx mori* without intestinal bacteria are susceptible to *Serratia piscatorum* and baculovirus; The intestinal microbiota of honeybees like those of *Bacillus* showed an antagonistic effect against *Paenibacillus* larvae; similarly, *Enterococcus* in the gut of *B. mori* can protect the host from some toxins and help to resist bacterial pathogens (Meister et al. 2009, Crotti et al. 2012, Grau et al. 2017, Li et al. 2020). Midgut bacteria in the larvae of a few species have also been implicated in promoting the insecticidal activity of *Bt* (Broderick et al. 2006). A recent study shows that the killing mechanism of *Bt* is through the intestinal epithelial lesions induced by a Cry toxin, which leads to the uncontrolled proliferation of midgut bacteria after entering the body cavity, and finally increases the mortality of the host (Caccia et al. 2016). However, less is known about the specific role of single midgut bacteria in mediating fall webworm–entomopathogen interactions.

Although HcNPV and *Bt* have been employed in the control of *H. cunea*, the role of intestinal bacteria, especially the dominant ones, in infection is still unclear. In the present study, bacteria from the midgut of the fourth instar larvae of *H. cunea* were isolated with culture-dependent method and identified by molecular biology and physiological and biochemical detection methods. The most dominant strains were screened to test their role in the infection of HcNPV and *Bt* against *H. cunea*.

## Materials and Methods

### Insects

*H. cunea* larvae were collected from Xiaohu Park, Huai’an District, Huai’an City, Jiangsu Province, China (33°53ʹN, 119°15ʹE). The larvae were transported to the laboratory and provided with fresh poplar leaves for feeding, which were replaced every 24 h until the adults mated and oviposited. The egg masses were collected and fumigated with 5% formalin for 15 min. Each egg mass was placed in a sterile petri dish and incubated in an environmental chamber under a 25 ± 2°C, 70% ± 5% RH, and a photoperiod of 16:8 (L:D) h. Upon hatching, the larvae (in groups of 100–150) were fed a fresh artificial diet (purchased from the Ecology and Nature Conservation Institute, Chinese Academy of Forestry).

Newly molted fourth instar larvae (*n* = 10) were transferred to sterile petri dishes and starved for 24 h. Individual larvae were then randomly selected and surface sterilized in 75% ethanol for 90 s, followed by rinsing three times in sterilized water. Each larva was dissected under aseptic conditions using dissection scissors and fine-tipped forceps. The intestine in its entirety was taken out and the midgut was separated and kept at −20°C until assigned to experiments. The midgut was transferred into a 1.5 ml centrifuge tube containing 0.5 ml of 10 mM PBS. Midgut in the tube was macerated with a plastic pestle, and vortexed at medium speed on ice for 30 s. After shaking, 100 μl homogenate was drawn from the tube and loaded into a new centrifuge tube for bacterial culture. Three tubes were prepared.

### Isolation and Identification of Predominant Midgut Bacteria

Homogenate in each tube was diluted to 10^−3^–10^−6^ dilutions with sterile water, and plated on lysogeny broth (LB) agar media. The plates were incubated in the dark at 25°C for 72 h; single colonies with different appearances, sizes, forms, elevations, edges, colors, and opacities were then picked out and cultured on fresh LB agar plates. Two single colonies with the largest and second largest numbers in the plates were recorded as dominant bacteria in the midgut (HcM3 and HcM7). The final pure colonies were enriched in LB media, mixed with 30% glycerol, and stored at −80°C.

The bacterial genome from pure isolates was extracted using a TIANamp Bacteria DNA Kit (Tiangen, Beijing, China) according to the manufacturer’s instructions. The DNA quality was assessed using a Nanodrop 2000C (Thermo Scientific, Waltham, MA, USA), and was visualized with 0.8% agarose gel electrophoresis. DNA samples were stored at −20°C until required.

Bacterial 16S rRNA gene was PCR amplified using universal primers: 27 F (5ʹ-AGAGTTTGATCCTGGCTCAG-3ʹ) and 1492 R (5ʹ-GGTTACCTTGTTACGAC TT-3ʹ) in an Eppendorf Mastercycler (Eppendorf, Hamburg, Germany) (Weisburg et al. 1991). The PCR reaction was carried out in 25 μl mixtures with 1 μl of DNA template, 12.5 μl of Phusion High-Fidelity PCR Master Mix (New England Biolabs, Ipswich, MA, USA), 0.5 μl of 10 μM forward and reverse primers, and 10.5 μl of ddH_2_O. The cycle conditions were as follows: initial denaturation at 94°C for 1 min, followed by 35 cycles of denaturation at 98°C for 10 s, annealing at 55°C for 30 s, elongation at 72°C for 2 min, and a final extension at 72°C for 10 min. The amplification was confirmed by electrophoresis on 1% agarose gel with DNA-safe stain, and all PCR products were sent to Sangon Biotech Co., Ltd (Shanghai, China) for sequencing.

The obtained sequences were aligned with those in the GenBank database of the National Center for Biotechnology Information (NCBI; http://blast.ncbi.nlm.nih.gov) using the BLAST algorithm and compared with the reference sequences (Shakarami et al. 2019). Phylogenetic analysis was carried out using the MEGA software (version 7.0), and the sequences were aligned using ClustalW software (version 2.0). The phylogenetic tree was constructed to show the relationships among isolates using maximum likelihood approach and assessed with 1,000 bootstrap replicates (Xu et al. 2015).

Traditional physiological and biochemical tests were also conducted to further identify the species of bacteria. Gram staining, capsule staining, and flagella staining were performed to observe the characteristics and shapes of bacteria under 100× magnification using an oil-immersion objective. The ranges of pH, temperature, and salt (NaCl) tolerance suitable for strain growth were determined. Other biochemical tests conducted were for activities of catalase, phenylalanine deaminase, urease, arginine dihydrolase, ornithine decarboxylase, and lysine decarboxylase. In addition, other tests included methyl red (MR), Voges–Proskauer (V–P), indole, and hydrogen sulfide (H_2_S). Furthermore, reduction of nitrate; hydrolyzation of starch, gelatin, and esculin; utilization of citrate and malonate; and carbon utilization of glucose, sucrose, maltose, lactose, D-xylose, D-galactose, L-rhamnose, trehalose, L-arabinose, raffinose, fructose, mannitol, sorbitol, and inositol were also assessed (Cheesbrough 2005, Huang et al. 2012).

### Establishment of a *H. cunea* Larvae Strain without Intestinal Bacteria

To eliminate intestinal bacteria in *H. cunea* larvae, the fourth instar larvae were fed a sterilized artificial diet soaked in a 500 μg/ml mixture of rifamycin, streptomycin, gentamicin, kanamycin, and ampicillin for 24 h. Subsequently, the larvae were fed with a normal sterilized artificial diet. After 72 h of antibiotic treatment, the intestines of the larvae were extracted according to the protocol already explained; culture-dependent (colony-forming assay, CFUs/ml) and culture-independent methods were used to ascertain the elimination of intestinal bacteria. The larvae without antibiotic treatment were used as control, and all the treatments were repeated thrice. The survival rate and pupation rate of control (*n* = 60) and antibiotic-treated larvae (*n* = 60) were determined to exclude the influence on bioassay results (Lin et al. 2015, Xia et al. 2020).

### Determination of Median Lethal Concentrations (LC50) of HcNPV and *Bt*

Toxicity tests were conducted using the diet surface overlay method (Wei et al. 2016, Singh et al. 2018). The HcNPV and *Bt* (NL-*Bt*2 strain) used in the tests were provided by the Chinese Academy of Forestry and the Microbiology Research Group of Nanjing Forestry University, respectively. The stock solution of HcNPV was diluted to five different concentrations: 1.0 × 10^3^, 1.0 × 10^4^, 1.0 × 10^5^, 1.0 × 10^6^, and 1.0 × 10^7^ PIBs/ml. Every 20 larvae were treated with one concentration, and each treatment was repeated three times (*n* = 60), while the untreated larvae were used as the controls. In the experiment, the larvae were treated in aseptic plastic petri dishes (35 mm in diameter), while each larva was placed in one dish. The sterilized artificial diet was made into round flakes (3 mm in diameter and 1 mm thick) by using a puncher and a blade, and each flake was mixed with 2 μl diluent of each of the five concentrations of HcNPV in different tubes. The dried flakes and the healthy fourth instar larvae of *H. cunea* (the strain without intestinal bacteria) with 8 h starvation were put into the petri dishes. The larvae were then reared in the climatic chamber at 25 ± 2°C with 70% ± 5% RH, and a photoperiod of 12:12 (L:D) h. After 24 h of feeding, the larvae were transferred to dishes with an untreated diet with daily diet renewal. The numbers of dead larvae at 24 h intervals were recorded until all larvae pupated. Depending upon the mortality of the control larvae, Abbott’s formula was used to correct the mortality data. The formula is


$$\begin{array}{l} Pct\;=\frac{Pt-Pc}{1-Pc}\times 100\% \end{array}$$

where *P*_*ct*_ is the corrected mortality, *P*_*t*_ is the treatment mortality, and *P*_*c*_ is the control mortality.

The cultured NL-*Bt*2 suspension was also diluted to the following five concentrations: 2.3 × 10^4^, 2.3 × 10^5^, 2.3 × 10^6^, 4.6 × 10^6^, and 2.3 × 10^7^ CFUs/ml. Every 36 larvae were treated with one concentration, and each treatment was repeated three times (*n* = 108), while the untreated larvae were used as the control. The toxicity test on *Bt* was performed as previously described for HcNPV.

### Bioassay of Dominant Bacteria in the Midgut of *H. cunea* Larvae

The bioassays were carried out using the diet surface overlay method by individually inoculating two dominant culturable bacterial strains (HcM3 and HcM7) into the intestine of *H. cunea* larvae devoid of any intestinal bacteria. The fresh artificial diet flake described above was smeared with 2 μl of each strain diluted to 10^6^ CFUs/ml. After 24 h of feeding, the larvae were transferred to dishes with an untreated diet with daily diet renewal. The successful inoculation and colonization of bacteria were detected by the culture-dependent and culture-independent methods described above. Assays with HcNPV and *Bt* were both divided into 6 groups consisting of *H. cunea* without intestinal bacteria (control), only containing HcM3, containing HcM7 that were fed with untreated diet, and *H. cunea* without intestinal bacteria, only containing HcM3, containing HcM7 that were fed with diets treated with HcNPV or *Bt*. In each group, 20 (HcNPV) or 30 (*Bt*) larvae were treated, and each treatment was repeated thrice. The preparation and treatment of the diet, and larval rearing were performed according to the above methods of toxicity tests, while LC_50_ of HcNPV and *Bt* were used to treat the diet_._ The numbers of dead larvae at 24 h intervals were recorded until all larvae pupated.

### Statistical Analysis

Results of colony forming assay from control and antibiotic treatment groups were evaluated using Student’s *t*-test, and the data are displayed as mean ± standard deviation (SD). Differences in the survival and pupation rate between the larvae of the control group and the antibiotic treatment group were evaluated using the chi-square test (Χ^2^ test). Probit analysis was performed to calculate LC_50_ and its 95% confidence limit (CL) of HcNPV and *Bt*. The differences in larval mortality among the different bioassay groups were determined using one-way ANOVA followed by Tukey’s HSD post hoc test. The mortality data were first arcsine square-root transformed for meeting normal distribution and homogeneity of variance. All analyses were performed using IBM SPSS Statistics (version 22.0), and the threshold for statistical significance was set as 0.05 (*P* < 0.05).

## Results

### Identification of Predominant Bacteria in the Midgut of *H. cunea* Larvae

Based on the morphological characteristics of colonies, a total of 15 strains of bacteria were isolated from the midgut of *H. cunea* larvae using the culture-dependent method and numbered (No.) from HcM1 to HcM15. The results of Gram staining, capsule staining, and flagella staining and the shapes of all strains were described (Supplementary Table S1). Two predominant bacterial strains, HcM3 and HcM7, were separately isolated and preserved according to the numbers of colonies.

**Fig. 1.**
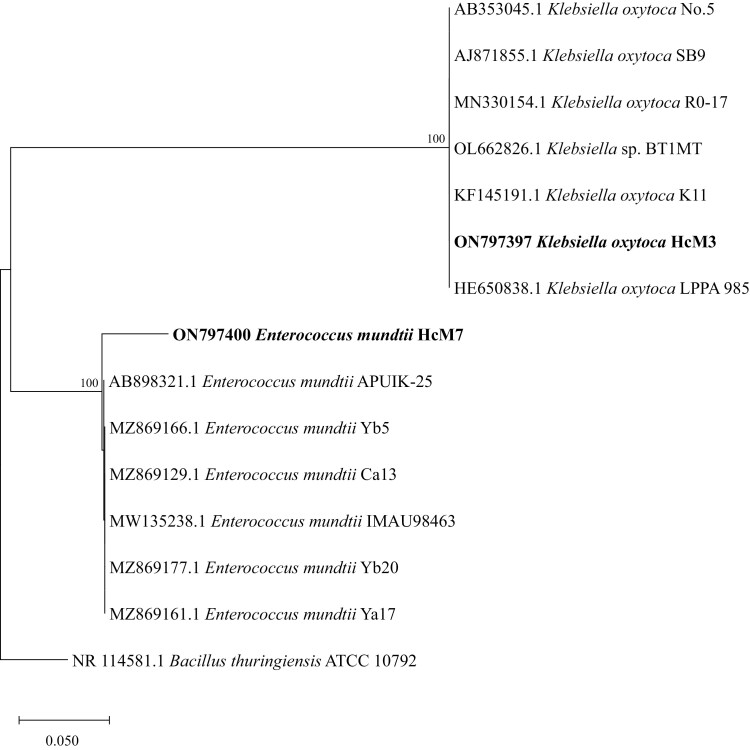
Phylogenetic tree of 16S rRNA gene sequences of dominant midgut-bacteria isolated from *H. cunea* larvae (1,000× bootstrapping).

The 16S rRNA gene sequences from two predominant isolates showed high similarities (≥98%) to the reference sequences BLAST searched using the GenBank database (GenBank accession numbers ON797397 and ON797400 corresponded to strains HcM3 and HcM7). A phylogenetic tree was constructed based on 16S rRNA gene sequences of strains HcM3 and HcM7, and representative taxonomically related species were identified using the maximum likelihood approach (Fig. 1). Sequence analysis indicated that two predominant isolates were homologous with two different genera, *Klebsiella* (HcM3) and *Enterococcus* (HcM7), and closely associated with *Klebsiella oxytoca* and *Enterococcus mundtii*.

Physiological and biochemical tests of two predominant isolates were conducted, and their characteristics are tabulated in Supplementary Tables S2 and 3. The isolates were identified and classified using Bergey’s bacterial identification manual and the Common Bacterial System Identification Manual, combined with the results of sequence analysis and characteristics of each strain (Buchanan and Gibbens 1984, Dong and Cai 2001). The results showed that two dominant bacterial strains were identified as *K. oxytoca* (HcM3) and *E. mundtii* (HcM7).

### Obtaining *H. cunea* Larvae Without Intestinal Bacteria

**Fig. 2.**
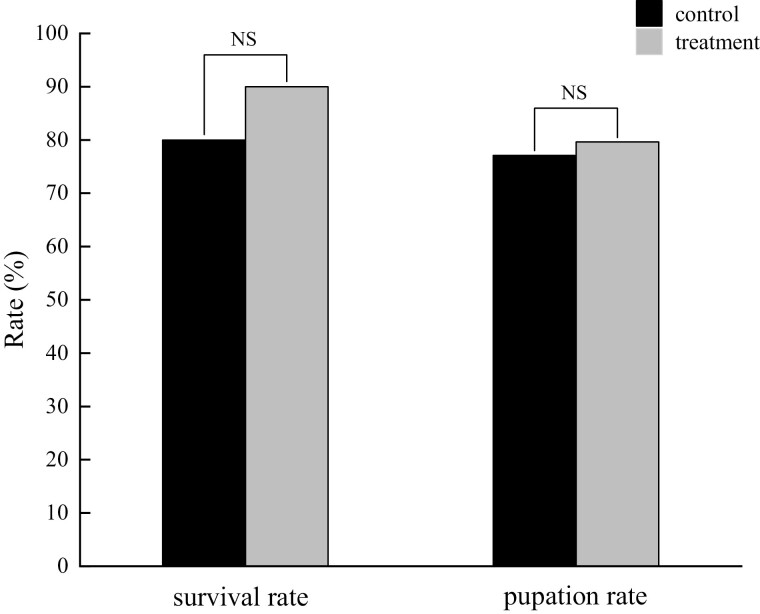
Survival and pupation rate of *H. cunea* larvae between antibiotic treatment and control. † NS: no statistical significance (Χ^2^ test, *P* > 0.05).

The results of colony forming assay and DNA gel electrophoresis showed significant difference between the control (6.7 × 10^7^ ± 1.0 × 10^6^ CFUs/ml) and antibiotic treatment (232.3 ± 13.2 CFUs/ml) groups (*t* = 237.407; df = 4; *P* < 0.05), which indicates that the intestinal bacteria of *H. cunea* larvae can almost be eliminated (Supplementary Fig. S1). The survival rates of *H. cunea* larvae were greater than or equal to 80%, and the pupation rates exceeded 75%. In addition, the survival rate (χ^2^ = 2.353; df = 1; *P* = 0.125) and pupation rate (χ^2^ = 0.097; df = 1; *P* = 0.755) were not significant between the control and antibiotic-treated groups (Fig. 2). The results show that the absence of intestinal bacteria did not affect survival and development of *H. cunea* larvae, and the bioassay tests were carried out smoothly.

### LC_50_ of HcNPV and *Bt*

The dose–mortality response of the fourth instar larvae of *H. cunea* to HcNPV and *Bt* are as detailed in Table 1. The LC_50_ value of HcNPV was 2.580 × 10^5^ PIBs/ml, and the value of *Bt* was 2.916 × 10^6^ CFUs/ml. The resulting LC_50_ values of HcNPV and *Bt* were used in subsequent bioassays.

**Table 1.** Dose-mortality response of *H. cunea* larvae to HcNPV and *Bt*

Treatment	Regression equation	*n*	Slope ± SE^*a*^	LC_50_ (95% CL)	χ^2^
HcNPV (PIBs/ml)	*y* = −3.465 + 0.640*x*	60	0.640 ± 0.067	2.580 × 10^5^ (1.431 × 10^5^ −4.858 × 10^5^)	5.191
*Bt* (CFUs/ml)	*y* = −8.163 + 1.263*x*	108	1.263 ± 0.101	2.916 × 10^6^ (1.094 × 10^6^ −8.171 × 10^6^)	13.575

^*a*^SE: standard error.

### Effects of Dominant Bacteria in the Midgut of *H. cunea* Larvae on the Toxicity of HcNPV and *Bt*

**Fig. 3.**
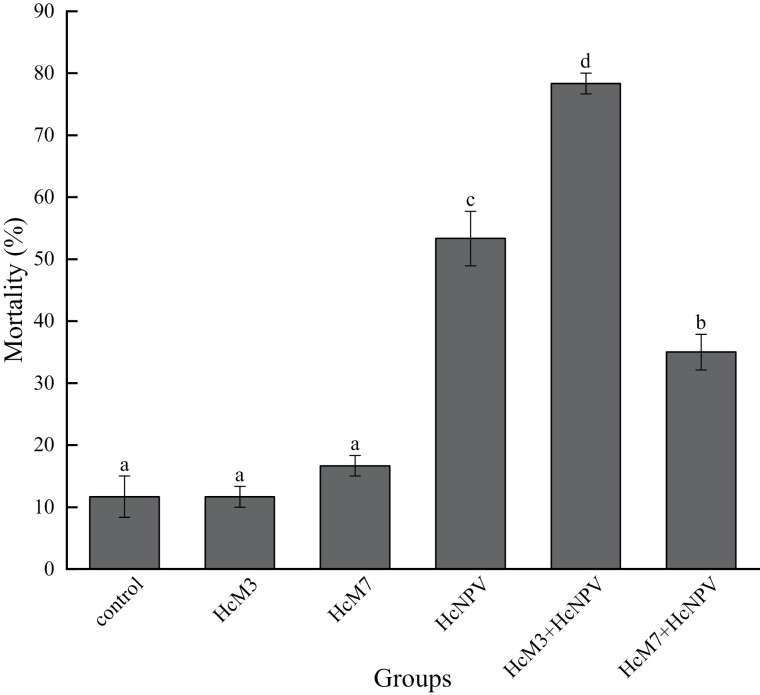
Mortality in different groups treated with HcNPV. † Different lowercase letters indicate significant differences among the groups (one-way ANOVA, Tukey’s HSD analysis, *P* < 0.05). The error bars are indicative of the standard error.

**Fig. 4.**
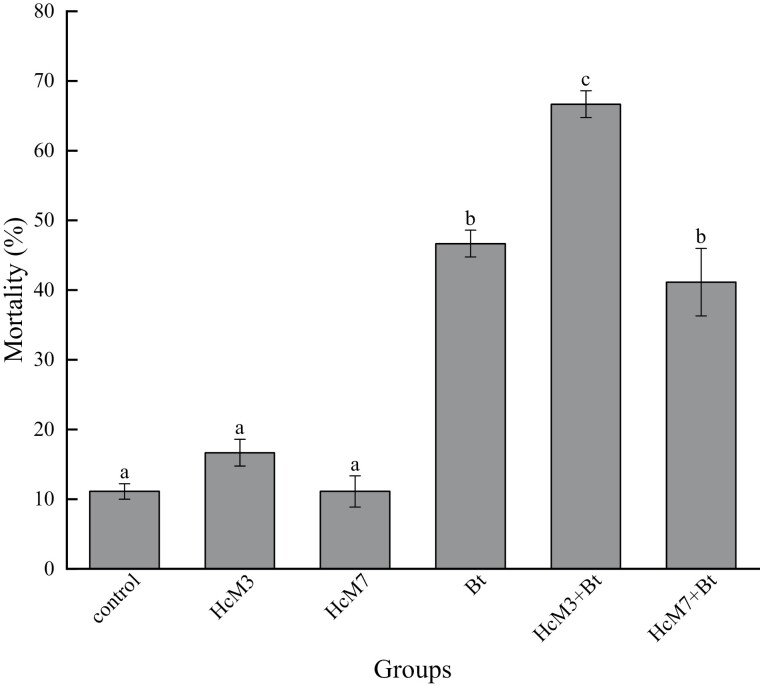
Mortality in different groups treated with *Bt.* † Different lowercase letters indicate significant differences among the groups (one-way ANOVA, Tukey’s HSD analysis, *P* < 0.05). The error bars are indicative of the standard error.

The mortality in different groups of *H. cunea* larvae that were treated with HcNPV and *Bt* are illustrated in Fig. 3 (F = 70.113; df = 5, 12; *P* < 0.001) and Fig. 4 (F = 70.011; df = 5, 12; *P* < 0.001). In the bioassay of HcNPV (Fig. 3), pairwise comparison showed no statistical significance in mortality among the control, *K. oxytoca*, and *E. mundtii* groups, and mortality was low in all three groups (<20%). These results indicate that the mortality of *H. cunea* larvae was not significantly influenced by inoculation of the dominant strains *K. oxytoca* or *E. mundtii*. The mortality of the larvae without intestinal bacteria treated with HcNPV was significantly higher than that of control, *K. oxytoca*, and *E. mundtii*. The mortality of *K. oxytoca* + HcNPV was almost 80% (78.33%), significantly higher than that of HcNPV, which indicates that the dominant strain *K. oxytoca* enhanced the toxicity of HcNPV. In contrast, the dominant strain *E. mundtii* significantly weakened the toxicity of HcNPV; however, the mortality (35%) was still higher than that in the control, *K. oxytoca*, and *E. mundtii*.

In the bioassay of *Bt* (Fig. 4), the results were similar to those of HcNPV. The mortality of *H. cunea* larvae was not affected by inoculation of either of the dominant strains, *K. oxytoca*, or *E. mundtii*. The mortality of larvae fed on the diet with *Bt* was significantly higher than that of control larvae and the larvae treated only with *K. oxytoca* or *E. mundtii*. The presence of the dominant strain *K. oxytoca* significantly strengthened the toxicity of *Bt* in *H. cunea* larvae, while the dominant strain *E. mundtii* neither strengthened nor weakened the toxicity of *Bt*.

## Discussion

In our study, both *E. mundtii* and *K. oxytoca* were isolated as the dominant bacterial strains from the midgut of *H. cunea* larvae, which was similar to *H. cunea* larvae separately fed on artificial diet and other host leaves (Wei et al. 2017, Deng et al. 2022). However, the composition and structure of intestinal microbiota of *H. cunea* larvae that feeds on artificial diet and plant leaves are different, and the diversity of bacterial community that feeds on artificial diet is lower (Deng et al. 2022). Therefore, our study only isolated strains from the gut of *H. cunea* larvae fed on artificial diet, which may not be widely representative, and more strains with similar functions have not been found. More tests are needed to prove that the strains isolated in the laboratory can play the same role when *H. cunea* feeds on the host leaves in the wild. *Enterococcus*, as the most abundant genus, was also found in the gut of *Brithys crini* (lily borer) and *Spodoptera littoralis* (cotton leafworm) (Mereghetti et al. 2017). In addition to being highly enriched in *S. littoralis*, *E. mundtii* was also detected in *Choristoneura fumiferana*, *Mythimna separata*, and *P. xylostella* (He et al. 2013, Landry et al. 2015, Ramya et al. 2016). In male *S. littoralis*, *Klebsiella* was dominant, and was also present in the abdomen of the moth *Calyptra thalictri* (Azambuja et al. 2005, Zaspel and Hoy 2008). *K. oxytoca* has been detected as the most important bacterial colonies in the genital chamber of beetle *Phyllophaga obsoleta* and in the guts of *Cleonus trivittatus* (Cleonus weevil), *Delia lupini* (Flower fly), and *Walshia miscecolorella* (Sweetclover root borer moth) (Rosete-Enriquez and Romero-López 2017, Itoh et al. 2018).

The present findings demonstrated that intestinal bacteria were almost eliminated in *H. cunea* larvae when fed with antibiotics, while the survival and pupation rates of larvae were unaffected. In *Spodoptera litura*, the larvae fed with antibiotics (streptomycin sulphate and rifampicin) treated diet showed that the elimination of intestinal bacteria did not affect their survival and development (Devi et al. 2022). Intestinal microorganisms play significant roles in host nutrition and digestion, and therefore the use of antibiotics may lead to altered insect intestinal microbiota and ultimately affect the fitness of insects (Thakur et al. 2016). After five antibiotics (rifampicin, ampicillin, tetracycline, streptomycin sulfate, and chloramphenicol) were used to eliminate gut bacteria in *P. xylostella* larvae, the growth and development of the larvae decreased, resulting in increased mortality, deformed prepupa, and hindered pupation and development into adults (Lin et al. 2015). The larval development duration of *Grapholita molesta* treated with the antibiotic (ciprofloxacin) was prolonged, and the adult longevity was shortened (Zhang et al. 2022). Similar results were also found in the antibiotic (tetracycline) treated *Pieris canidia* (Wang et al. 2020b). Furthermore, the hindgut symbionts of termites markedly influenced the survival of *Periplaneta americana*, and the elimination of bacteria reduced their fecundity (Cruden and Markovetz 1987). However, most of the bacteria in these studies were involved in hydrolysis of carbohydrates, such as degradation of lignocellulose, and providing glucose and fatty acids as an energy source to the hosts (Genta et al. 2006, Visôtto et al. 2009, Prem Anand et al. 2010). In *Rhynchophorus ferrugineus* (red palm weevil), for instance, the administration of antibiotics was not found to significantly affect body weight gain in larvae but dramatically decreased the concentration of hemolymph and glucose (Muhammad et al. 2017). The weight and body length of silkworms treated with vancomycin and chloramphenicol did not change significantly, but their feeding behavior considerably increased after antibiotic exposure (Li et al. 2020). On the contrary, an increase in tetracycline concentration in the diet did not significantly increase the mortality of *A. gemmatalis* but accelerated the development rate and increased pupa weight (Visôtto et al. 2009). Significantly higher larval mortality of *Spodoptera litura* was documented for a diet without antibiotics than for a diet treated with streptomycin sulfate, for which the pupation rate was significantly higher (Thakur et al. 2016). Similarly, increasing the dose of antibiotics could increase the survival rate of *Galleria mellonella* larvae infected with *Staphylococcus aureus*, and low concentrations of antibiotics did not adversely affect the survival of *Pimpla turionellae* larvae (Büyükgüzel and Yazgan 2001, Desbois and Coote 2011). These opposing results may be due to the significant inhibition of nonsymbiotic intestinal bacteria in insects treated with antibiotics, which is more beneficial to their survival and development compared with insects without treatments (Visôtto et al. 2009). In our artificial diet for raising *H. cunea* larvae, sugar and other energy substances were added. These substances can be absorbed and utilized through simple hydrolysis processes without the participation of intestinal bacteria, which may be an important reason for the elimination of intestinal bacteria not affecting the survival and pupation of larvae.

We have shown that both HcNPV and *Bt* are pathogenic in *H. cunea* larvae without intestinal bacteria. Raymond et al. (2009) and Johnston and Crickmore (2009) also proved that gut bacteria are not required for the pathogenicity of *Bt* in *P. xylostella* and *Manduca sexta*. More interestingly, the inoculation of *K. oxytoca* greatly increased the susceptibility of *H. cunea* larvae to HcNPV and *Bt*, while the inoculation of *E. mundtii* reduced the mortality of larvae infected with HcNPV and *Bt*. Therefore, in *H. cunea* larvae, the presence of some intestinal bacteria may increase the risk of death from infectious pathogens, while others protect the host from pathogens. Broderick et al. (2006, 2009) found that administration of antibiotics reduced larval mortality due to *Bt* in five different lepidopteran species, and the toxicity of *Bt* was restored in four of these after inoculating with *Enterobacter* sp. NAB3. The mortality of *H. armigera* larvae reared on diets with four antibiotics was significantly lower than that without antibiotics (Paramasiva et al. 2014). These results suggest that the gut microbiota commonly influenced the pathogenicity of *Bt*. In contrast, Raymond et al. (2009) and van Frankenhuyzen et al. (2010) explained that the weakening of *Bt* pathogenicity was due to past antibiotic exposure rather than the removal of intestinal microbiota. Nevertheless, Broderick et al. (2009) deduced that antibiotics were unlikely to affect *Bt* pathogenicity because they found similar levels of *Bt* (CFUs) in the intestines of the larvae fed antibiotics or reared without antibiotics and that both *E. coli* and *Enterobacter* sp. NAB3 could proliferate in antibacterial treated larvae. In our study, *Bt* and HcNPV were fed after colonization of *K. oxytoca* or *E. mundtii* in *H. cunea* larvae to rule out the effect of antibiotics on *Bt* or HcNPV. In *L. dispar* and *C. fumiferana* inoculated with *Bt*, the mortality of larvae without midgut bacteria was always higher than that of larvae with midgut bacteria, implying that the intestinal microorganisms could protect the host from pathogens (Raymond et al. 2009, van Frankenhuyzen et al. 2010). Therefore, the research to date suggests that the influence of intestinal bacteria on the pathogenicity of pathogens may be two-sided, which is consistent with our results.

*Enterococcus*, as the dominant genus of Lepidoptera, protects the host from toxins. In the larvae of *L. dispar*, *S. litura*, *G. mellonella,* and *M. sexta*, *Enterococcus* was found to reduce the insecticidal activity of *Bt* (Broderick et al. 2004, Johnston and Crickmore 2009). The resistance of *Tribolium castaneum* larvae against *Bt* was improved after feeding with the isolate or the supernatant of *E. mundtii* (Grau et al. 2017). *E. mundtii* can form biofilm-like structures in the intestine of its insect host to provide protection (Vilanova et al. 2016). In addition, Shao et al. (2017) found that *E. mundtii* secreted an antimicrobial peptide in the gut lumen of *S. littoralis*, creating an additional chemical barrier against pathogens. Previous work has shown that *Enterococcus* bacteria can produce acetate, resulting in the decrease in pH of intestinal digestive juice (Mead et al. 1988). In our study, the MR test of *E. mundtii* was positive, indicating that *E. mundtii* could also secrete acidic substances to reduce intestinal pH. As *Bt* and HcNPV are toxic only under alkaline conditions, the decrease of intestinal pH can directly weaken the pathogenicity of these two pathogens. However, the protective effect of *E. mundtii* on *H. cunea* larvae treated with *Bt* was not significant, which may be attributable to competition for nutrition between *Bt* and *E. mundtii* (Takatsuka and Kunimi 2000). In contrast, the effects of *Klebsiella,* as intestinal bacteria, on insect fitness have rarely been reported. Nonetheless, *K. oxytoca* has attracted our attention because of its significant enhancement effects on HcNPV and *Bt*. We found that the MR test of *K. oxytoca* was negative while the V-P test was positive. This suggests that *K. oxytoca* further decarboxylates pyruvate into acetoin with the production of 2,3-butanediol instead of decomposing into acidic substances (Chen et al. 2014). *Klebsiella* species have been widely applied for the biological production of 2,3-butanediol because of their high productivity (Syu 2001). As early as 1906, *Klebsiella pneumoniae* (*Aerobacter aerogenes*) was employed to produce 2,3-butanediol, and then *K. oxytoca* was also used for production (Yang et al. 2007). It has been proved that 2,3-butanediol has potential applications in the manufacture of inks, perfumes, plasticizers, wetting agents, fumigants, softeners, and explosives and also been used as a carrier for pharmaceuticals (Xiu and Zeng 2008). We speculate that these properties of *K. oxytoca* are conducive for rapid diffusion of HcNPV and *Bt* without affecting the intestinal pH of *H. cunea* larvae, which increases the mortality.

Although we have separately tested the enhancement and weakening effect of HcNPV and *Bt* by two intestinal dominant bacteria, we have not observed the effect of inoculating two intestinal bacteria at the same time. Further experiments are needed to confirm these hypotheses and to identify other possible influencing factors. However, It is worth noting that the enhanced insecticidal activity of *K. oxytoca* on HcNPV and *Bt* provides a reference and theoretical basis for future biological control of *H. cunea* by intestinal bacteria.

## Supplementary Material

iead009_suppl_Supplementary_Material
