# Supplementary material for: Effects of midgut bacteria in Hyphantria cunea (Lepidoptera: Erebidae) on nuclear polyhedrosis virus and Bacillus thuringiensis (Bacillales: Bacillaceae)
Source: J Insect Sci. 2023 Mar 14;23(2):1. doi: 10.1093/jisesa/iead009 (PMC10011879; doi:10.1093/jisesa/iead009)
Supplement: iead009_suppl_Supplementary_Material [file iead009_suppl_supplementary_material.doc]

**TABLE S1** Characteristics and colonial morphology of isolated strains

| Strain No. | Gram stain | Capsule stain | Flagella stain | Strain shapes | [Colonial](../../../../D:/Dict/7.5.2.0/resultui/dict/%3Fkeyword=colonial)[morphology](../../../../D:/Dict/7.5.2.0/resultui/dict/%3Fkeyword=morphology) |
| --- | --- | --- | --- | --- | --- |
|
| HcM1 | G+ | - | - | rod | pink, round, smooth, convex, opaque, with neat edges |
|
| HcM2 | G+ | - | + | sphere | pale yellow, round, thick, convex, translucent, with halo edges |
|
| HcM3 | G- | + | - | rod | white, round, smooth, convex, transparent, with uneven edges |
|
| HcM4 | G+ | - | - | sphere | pale white, round, smooth, convex, lustrous, with transparent edges |
|
| HcM5 | G+ | - | + | rod | orange yellow, round, rough, convex, opaque, with halo edges |
|
| HcM6 | G+ | - | - | sphere | yellow, round, thick, smooth, opaque, with halo, neat edges |
|
| HcM7 | G+ | - | - | sphere | milky white, round, smooth, convex, lustrous, with transparent edges |
|
| HcM8 | G+ | - | - | rod | orange yellow, irregular, smooth, flat, shiny, translucent, with neat edges |
|
| HcM9 | G+ | - | - | short rod | [m](http://dict.youdao.com/w/creamy white/" \l "keyfrom=E2Ctranslation)ilky white, round, smooth, slightly convex in the center, opaque, with regular edges |
|
| HcM10 | G+ | - | - | sphere | grayish white, round, rough, convex, with uneven edges |
|
| HcM11 | G+ | - | - | sphere | dark yellow, round, smooth, convex, opaque, with neat edges |
|
| HcM12 | G+ | - | - | sphere | milky white, round, smooth, convex, opaque, with neat edges |
|
| HcM13 | G+ | - | - | sphere | grayish white, round, smooth, convex, lustrous, with neat edges |
|
| HcM14 | G+ | - | + | rod | milky white, round, smooth, convex, opaque, with neat edges |
|
| HcM15 | G+ | - | + | rod | milky white, round, smooth, convex, lustrous, with neat edges |
|

† Symbols: G+ gram-positive; G- gram-negative; + positive; − negative.

**TABLE S2** Physiological characteristics of 15 isolates

| Strain No. | pH | | | | | Temperature (°C) | | | | | | NaCl concentration (%) | | | | |
| --- | --- | --- | --- | --- | --- | --- | --- | --- | --- | --- | --- | --- | --- | --- | --- | --- |
|  | 2 | 5 | 7 | 9 | 11 | 4 | 15 | 28 | 37 | 45 | 60 | 1 | 2 | 5 | 7 | 10 |
| HcM3 | - | + | +++ | ++ | - | - | + | +++ | ++ | - | - | +++ | ++ | ++ | + | - |
| HcM7 | - | + | +++ | ++ | - | - | + | ++ | +++ | + | - | +++ | ++ | ++ | + | - |

† Symbols: + positive; − negative. More “+” means more suitable for strain growth.

**TABLE S3** Biochemical characteristics of 15 isolates

| Characteristics | Strain No. | |
| --- | --- | --- |
|  | HcM3 | HcM7 |
| Catalase | + | + |
| Phenylalanine deaminase | - | - |
| Urease | - | - |
| Arginine digydrolas | - | + |
| Ornithine decarboxylase | - | + |
| Lysine decarboxylase | + | - |
| MR test | - | + |
| V-P test | + | + |
| Indole test | + | - |
| H_2_S test | - | + |
| Nitrate reduction | + | + |
| **Hydrolysis of** |  | |
| Starch | - | + |
| Gelatin | - | + |
| Esculin | + | + |
| **Assimilation of** |  | |
| Glucose | + | - |
| Sucrose | + | + |
| Maltose | + | + |
| Mannitol | + | + |
| Sorbitol | + | - |
| Lactose | + | + |
| D-xylose | + | + |
| D-galactose | + | + |
| L-rhamnose | + | - |
| Trehalose | + | + |
| L-arabinose | + | + |
| Raffinose | + | + |
| Fructose | + | - |
| Inositol | + | + |
| Citrate | + | + |
| Malonate | + | + |

† Symbols: + positive; − negative.


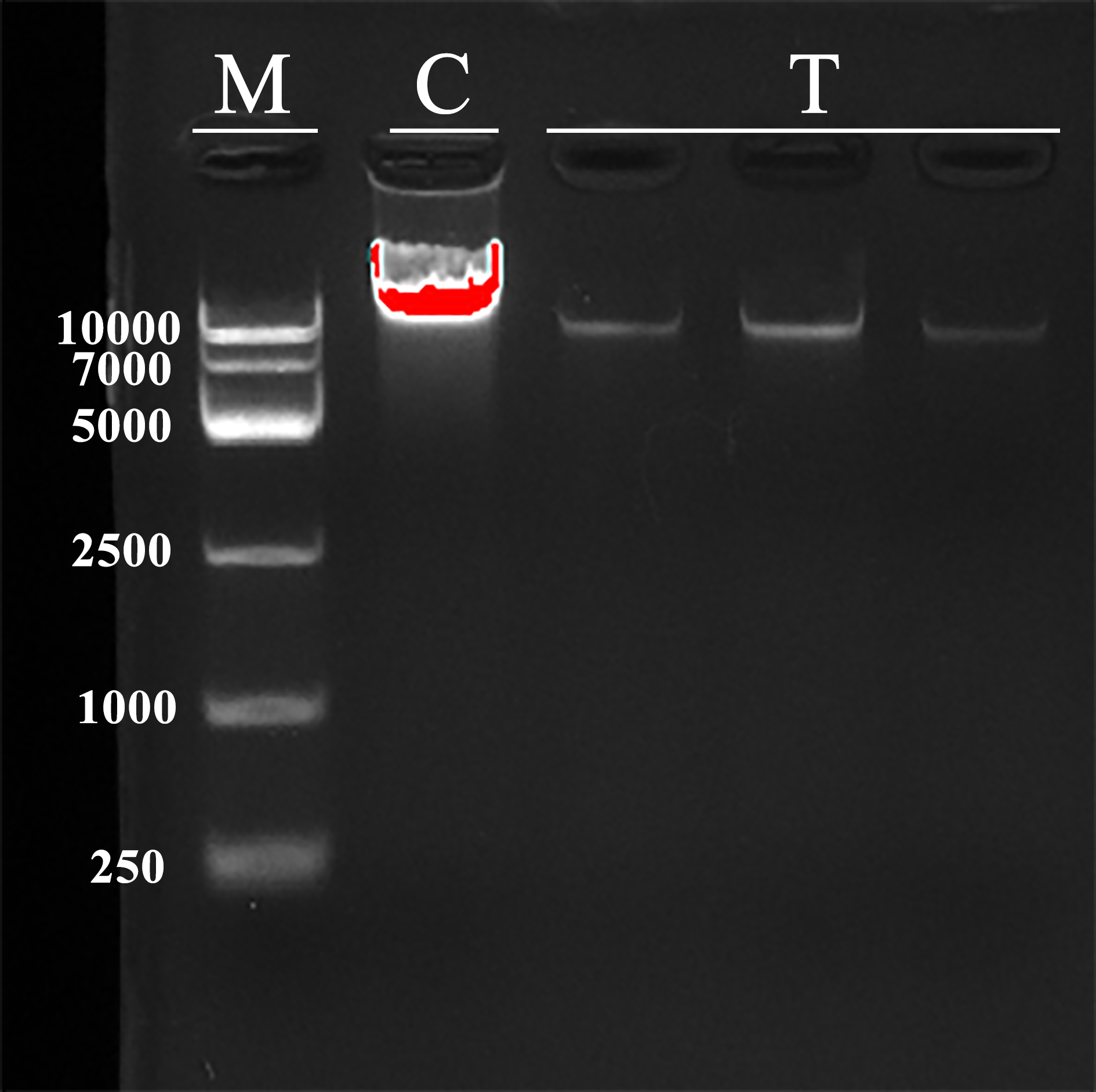


**FIGURE S1** DNA gel electrophoresis of *H. cunea* larvae with antibiotic treatment and control

† M: marker (bp), C: control, T: antibiotic treatment.
